# Genome Organization, Phylogenies, Expression Patterns, and Three-Dimensional Protein Models of Two Acetylcholinesterase Genes from the Red Flour Beetle

**DOI:** 10.1371/journal.pone.0032288

**Published:** 2012-02-16

**Authors:** Yanhui Lu, Yuan-Ping Pang, Yoonseong Park, Xiwu Gao, Jianxiu Yao, Xin Zhang, Kun Yan Zhu

**Affiliations:** 1 Department of Entomology, China Agricultural University, Beijing, People's Republic of China; 2 Computer-Aided Molecular Design Laboratory, Mayo Clinic, Rochester, Minnesota, United States of America; 3 Department of Entomology, Kansas State University, Manhattan, Kansas, United States of America; Weizmann Institute of Science, Israel

## Abstract

Since the report of a paralogous acetylcholinesterase (AChE, EC3.1.1.7) gene in the greenbug (*Schizaphis graminum*) in 2002, two different AChE genes (*Ace1* and *Ace2*) have been identified in each of at least 27 insect species. However, the gene models of *Ace1* and *Ace2*, and their molecular properties have not yet been comprehensively analyzed in any insect species. In this study, we sequenced the full-length cDNAs, computationally predicted the corresponding three-dimensional protein models, and profiled developmental stage and tissue-specific expression patterns of two *Ace* genes from the red flour beetle (*Tribolium castaneum*; *TcAce1* and *TcAce2*), a globally distributed major pest of stored grain products and an emerging model organism. *TcAce1* and *TcAce2* encode 648 and 604 amino acid residues, respectively, and have conserved motifs including a choline-binding site, a catalytic triad, and an acyl pocket. Phylogenetic analysis show that both *TcAce* genes are grouped into two insect *Ace* clusters and *TcAce1* is completely diverged from *TcAce2*, suggesting that these two genes evolve from their corresponding *Ace* gene lineages in insect species. In addition, *TcAce1* is located on chromosome 5, whereas *TcAce2* is located on chromosome 2. Reverse transcription polymerase chain reaction (PCR) and quantitative real-time PCR analyses indicate that both genes are virtually transcribed in all the developmental stages and predominately expressed in the insect brain. Our computational analyses suggest that the TcAce1 protein is a robust acetylcholine (ACh) hydrolase and has susceptibility to sulfhydryl agents whereas the TcAce2 protein is not a catalytically efficient ACh hydrolase.

## Introduction

Acetylcholinesterase (AChE, EC3.1.1.7) is an essential enzyme at the synapses of cholinergic neurons in the central and peripheral nervous systems in all animals. It catalyzes the hydrolysis of the neurotransmitter acetylcholine (ACh), thus terminating neurotransmission. AChE has long been of academic and industrial interest and studied extensively at the biochemical, biophysical, and molecular levels in mammals because this enzyme is a target of palliative Alzheimer drugs, nerve agents, and insecticides [1]. In insects, AChE has also been extensively studied because it serves as the target site for organophosphate and carbamate insecticides, and involves in insecticide resistance known as target-site insensitivity [2]–[8].

The first insect AChE gene (*Ace*) was sequenced from *Drosophila melanogaster* in 1986 [9]. After the first *Ace* paralogous gene was reported in the greenbug (*Schizaphis graminum*) in 2002 [10], the *D. melanogaster Ace* gene was designated as *Ace* orthologous gene. It is now clear that *D. melanogaster* has only one *Ace* gene as confirmed by its genome sequence [11], whereas most other insect species have two different *Ace* genes (*i.e.*, *Ace1* and *Ace2*) [12]. *Ace1* commonly refers to the *Ace* paralogous (AP-*Ace*) gene and *Ace2* the *Ace* orthologous (AO-*Ace*) gene in relation to the *D. melanogaster Ace* [8].

To date, the cDNAs encoding AChEs have been sequenced from at least 43 insect species. Among them, both *Ace1* and *Ace2* have been reported from each of 27 species, including *Bombyx mandarina* (GenBank accession numbers: EU262633 for *BmAce1* and EU262632 for *BmAce2*); *Sitobion avenae* [13]; *Rhopalosiphum padi* [13]; *Anopheles gambiae* [14]; *Liposcelis decolor* (GenBank accession numbers: FJ647186 for *LdeAce1* and FJ647187 for *LdeAce2*); *Orchesella villosa* [15]; *Liposcelis entomophila* (GenBank accession numbers: EU854149 for *LeAce1* and EU854150 for *LeAce2*); *Blattella germanica* [16]; *Bemisia tabaci* [17]; *Culex pipiens quinquefasciatus* (GenBank accession numbers: XM_001847396 for *CqAce1* and XM_001842175 *CqAce2*); *Bombyx mori* [18]; *Acyrthosiphon pisum* (GenBank accession numbers: XM_001948618 for *ApAce1* and XM_001948953 for *ApAce2*); *Nasonia vitripennis* (GenBank accession numbers: XM_001600408 for *NvAce1* and XM_001605518 for *NvAce2*); *Pediculus humanus corporis* [19]; *Cydia pomonella* [20]; *Helicoverpa assulta* [21]; *Aedes albopictus* (GenBank accession numbers: AB218421 for *AaAce1* and AB218420 for *AaAce2*); *Aphis gossypii* [22]; *Culex tritaeniorhynchus* [23]; *Myzus persicae* [24]; *Culex pipiens* [25]; *Plutella xylostella* [26]–[28]; *Chilo suppressalis* [29]; *Pediculus humanus capitis* [19]; *Aedes aegypti* [30]; *Liposcelis bostrychophila* (GenBank accession numbers: FJ647185 for *LbAce1* and EF362950 for *LbAce2*) and *Alphitobius diaperinus* [31]. The remaining insect species may also have two *Ace* genes but only one *Ace* gene (*Ace1* or *Ace2*) has been documented.

The existence of two *Ace* genes in insects has attracted much attention to the study of their functions, particularly their roles in insecticide resistance [8], [32] and as targets for developing new insecticides [12], [33]–[37]. Beetles (coleopterans) are the most evolutionarily successful metazoans, accounting for 25% of all known animal species, far more than any other taxonomic orders [31]. Despite the diversity and economic importance of coleopterans, *Ace* genes have been reported from only two species: *Leptinotarsa decemlineata* [5], [6] and *Alphitobius diaperinus* [31]. Although *T. castaneum* (the red flour beetle) is one of the most notorious stored grain pests in the world and is now regarded as an emerging model organism, its *Ace* genes were only predicted from genomic sequence and detailed information on these genes has been limited.

In this paper, we report two *Ace* genes from *T. castaneum*. Our study of the two genes focuses on the genome organization, three-dimensional (3D) protein models, phylogenies, and expression patterns of the two genes at different developmental stages of the insect, in an effort to better understand the functions of the two genes and obtain insights into better strategies for insect pest control.

## Results

### AChE cDNA and deduced amino acid sequences

**Figure 1:**
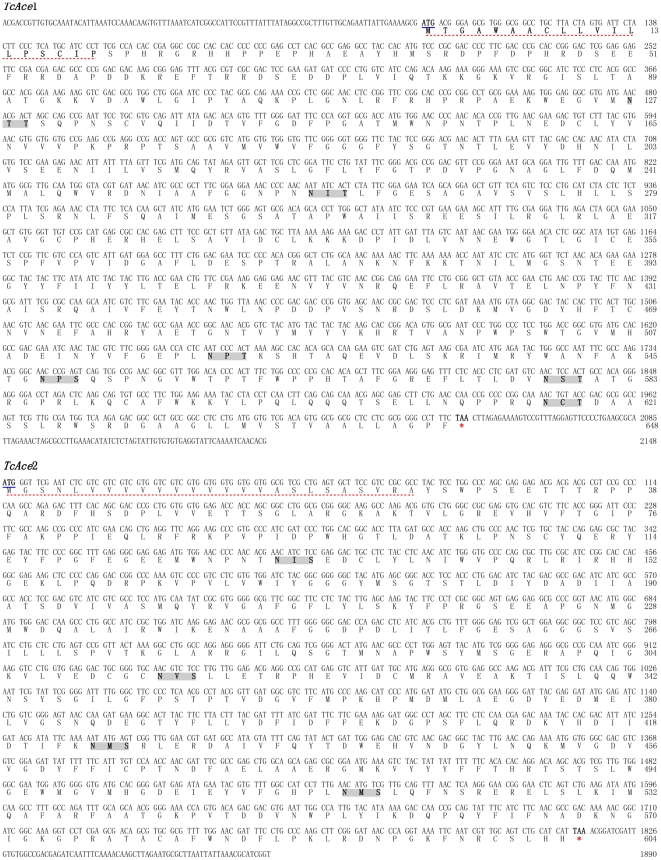
The cDNA and deduced amino acid sequences of two *Ace* genes from *Tribolium castaneum*. The amino acid sequences were numbered from the start of the mature proteins. The start codon ATG were bold and underlined, and the stop codon TAA at the end of the coding region were bold and marked with asterisks. The putative signal peptides of the deduced amino acid sequences were underlined with red dots. Potential N-linked glycosylation sites were bold and shaded. The sequences were deposited in the GenBank (accession numbers: HQ260968 for *TcAce1* and HQ260969 for *TcAce2*).

Based on the predicted sequences of two *T. castaneum Ace* genes in NCBI (XM_968369 and XM_965681), we designed specific primers (Table 1) to determine the full-length cDNAs of the two genes from the brain of *T. castaneum*. Each of several polymerase chain reaction (PCR) primer pairs was able to generate overlapping fragments for each gene and it was then possible to assemble them into its full-length cDNA of the protein coding region. The two deduced amino acid sequences show significant similarities to AChE1 (AP-AChE) and AChE2 (AO-AChE) proteins of other insects in GenBank according to our BLASTP analysis. Therefore, the two *T. castaneum Ace* genes are named *TcAce1* (AP-*Ace*) and *TcAce2* (AO-*Ace*), and their protein products are named TcAce1 and TcAce2, respectively. The *TcAce1* cDNA contains 2148 base pairs (bp) and has an open reading frame (ORF) of 1944 bp, encoding a protein of 648 amino acid residues, whereas the *TcAce2* cDNA contains 1,890 bp and has an ORF of 1,812 bp, encoding a protein of 604 residues. However, we were not able to obtain the 5′-untranslated region (5′-UTR) of the *TcAce2* cDNA (Fig. 1).

**Table 1:** PCR primers used to amplify cDNA sequences of both *TcAce1* and *TcAce2* genes and to analyze their gene expressions.

Primer name	Sequence (5′-3′)	Tm (°C)	Product size (bp)	Location^a^
*PCR for cDNA sequences*				
*TcAce*1-F^b^	CGGCCTGCTTACTAGTGATTCTAC	60.66	1714	17-1730
*TcAce*1-R	ACATCGAGGGTGAGAAACTCC	60.50		
*TcAce*1S-F	CAACGACCGTTGTGCAAATA	60.56	320	N/A-219
*TcAce*1S-R	CAGGGGATCATCTTCGGAGT	61.39		
*TcAce*1E-F	CGTTTGGACACCCACTTTCT	60.01	402	1665-N/A
*TcAce*1E-R	GTCGTGTTGATTTTGAATACCTCAC	60.99		
*TcAce*2-F	AGACCTCATCACGCTGTTTG	55.2	1034	750-1783
*TcAce*2-R	CTGGGTTATCCCGAAGCTTG	61.86		
*TcAce*2S-F	GTCGTAGAGGCGTCGTCGT	61.44	1014	N/A-895
*TcAce*2S-R	TCTCCCCCGACATGTAACTC	59.93		
*TcAce*2E-F	AACCAGTGACAGACGACGTG	59.78	267	1627-N/A
*TcAce*2E-R	CGCAACCGATGCGTTTAATA	61.86		
*Quantitative real-time PCR*				
*TcAce*1(Q)-F	CCGTTCGTCCCAGTCATTG	55.3	121	1069-1189
*TcAce*1(Q)-R	AGTAGTAGCCTTCTTCTGTGTTAG	55.4		
*TcAce*2(Q)-F	AGACCTCATCACGCTGTTTG	55.2	179	750-928
*TcAce*2(Q)-R	CCTCCACCAGGACCTTCC	54.9		
*TcRps*3-F	CCGTCGTATTCGTGAATTGAC	54.8	130	279-408
*TcRps*3-R	TCTAAGAGACTCTGCTTGTGC	54.7		

a Product location refers to the PCR fragment corresponding to the *Ace* gene nucleotide sequence of *T. castaneum* from NCBI database (*TcAce*1: XM_968369; *TcAce*2: XM_965681). N/A refers to the sequence based on the genomic sequence in Beetlebase (http://beetlebase.org/).

b F and R refer to forward and reverse primers, respectively.

**Figure 2:**
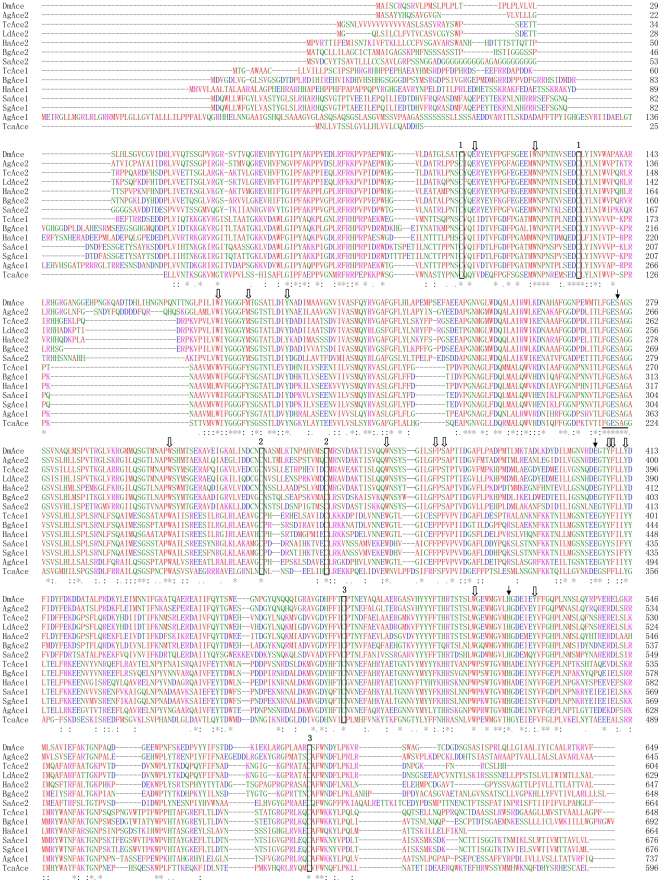
Alignment of deduced AChE protein sequences encoded by TcaAce (CAA27169, *Torpedo californica* Ace); TcAce1 (HQ260968, *Tribolium castaneum* Ace1, this paper); TcAce2 (HQ260969, *T. castaneum* Ace2, this paper); SaAce1 (AY819704, *Sitobion avenae* Ace1); SaAce2 (AY707319, *S. avenae* Ace2); DmAce (X05893, *Drosophila melanogaster* Ace); AgAce1 (XM_321792, *Anopheles gambiae* Ace1); AgAce2 (BN000067, *A. gambiae* Ace2); BgAce1 (DQ288249, *Blattella germanica* Ace1); BgAce2 (DQ288847, *B. germanica* Ace2); HaAce1 (DQ001323, *Helicoverpa assulta* Ace1); HaAce2 (AY817736, *H. assulta* Ace2); SgAce1 (AF321574, *Schizaphis graminum* Ace1) and LdAce2 (L41180, *Leptinotarsa decemlineata* Ace2). Numbering of the amino acid sequences was from the N-terminus of mature proteins. Identical amino acids were indicated by asterisks and conservative substitutions by dots. The catalytic triad residues were marked with arrowhead. The number 1, 2, 3 on the boxed amino acids indicated the residues forming intramolecular disulfide bonds. The positions of aromatic residues lining the active site gorge in *T. californica* AChE were marked with block arrows. The cholinesterase signature sequence was underlined.

*TcAce1* and *TcAce2* belong to typical *Ace1*- and *Ace2*-type genes, respectively, as judged by their sequence similarities with other known insect *Ace*s (Fig. 2). The deduced amino acid sequences (TcAce1 and TcAce2) of *TcAce1* and *TcAce2* exhibit six and four *N*-glycosylation sites (N-X-S or N-X-T) [38], respectively (Fig. 1). Predicted isoelectric points (pI) and molecular masses of TcAce1 and TcAce2 are 6.58 and 5.39, and 72.81 and 68.15 kDa, respectively (http://www.scripps.edu/~cdputnam/protcalc.html). Both TcAce1 and TcAce2 are predicted to contain a cleavable signal peptide, which suggests that these proteins can be secreted and function in an extracellular environment. Both proteins have a C-terminal Cys residue (C617 in TcAce1 and C600 in TcAce2) that is likely to form an intermolecular disulfide bond. According to the analysis using PredGPI (http://gpcr.biocomp.unibo.it/predgpi/), TcAce1 appears to contain a GPI-anchor, which is linked to the C-terminal residue after a proteolytic cleavage at the ω site (D619 in TcAce1), whereas TcAce2 doesn't seem to contain a GPI-anchor. Both proteins have relatively high sequence identity (47% for TcAce1 and 59% for TcAce2) to the C-terminal residues 526–543 (*i.e.*, QTCAFWNRFLPKLLSAT) of the recombinant mouse AChE (mAChE) [39], [40]. This level of sequence identity is identical or higher than the corresponding sequence identity (47%) between mAChE and *D. melanogaster* AChE (DmAChE). All these suggest that residues 589–605 in TcAce1 and residues 578–594 in TcAce2 are likely responsible for the formation of a dimeric four-helix bundle at the C-terminus as seen in mAChE [40] and DmAChE [41].

### Chromosomal locations of *TcAce1* and *TcAce2*

**Figure 3:**
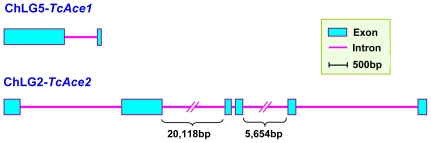
Schematic diagram of the organization of two *Ace* genes from *Tribolium castaneum*. The full lengths of the two genomic sequences were 2,986 bp for *TcAce1* and 32,243 bp for *TcAce2*. Genome structure showed that two different *Ace* genes in *T. castaneum* located on different chromosomes. *TcAce1* located on chromosome 5 and *TcAce2* on chromosome 2. *TcAce1* has two exons and one intron, whereas *TcAce2* has six exons and five introns.

The exon-intron organizations of *TcAce1* and *TcAce2* were revealed by comparisons of the full-length cDNAs with their corresponding genomic sequences (http://beetlebase.org/). The lengths of *TcAce1* and *TcAce2* genomic DNA sequences are 2,986 bp and 32,243 bp, respectively. Genome structure analysis shows that the two genes are located on different chromosomes of *T. castaneum*; *TcAce1* is located on chromosome 5, whereas *TcAce2* on chromosome 2. *TcAce1* has two exons and one intron, whereas *TcAce2* has six exons and five introns (Fig. 3).

### Phylogenetic relationship of *T. castaneum* AChE to other AChEs

**Figure 4:**
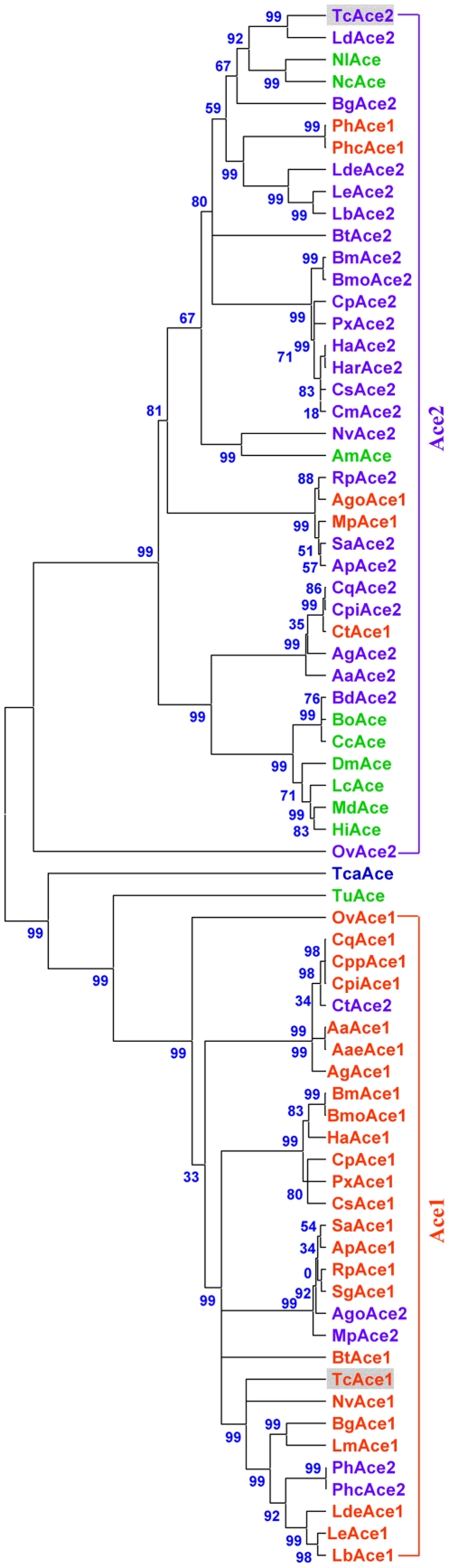
Rooted phylogenetic tree of deduced *Ace* amino acid sequences from the Pacific electric ray (*Torpedo californica*), two-spotted spider mite (*Tetranychus urticae*) and 43 insect species constructed by the neighbor-jointing method. The name is made up of a species abbreviation (first letter of the genus followed by the first one or two letters of the specific name). Sequences used: TcaAce (CAA27169, *T. californica* Ace); TcAce1 (HQ260968, *Tribolium castaneum Ace1*, this paper); TcAce2 (HQ260969, *T. castaneum Ace2*, this paper); BmAce1(EU262633, *Bombyx mandarina* Ace1); BmAce2 (EU262632, *B. mandarina* Ace2); SaAce1 (AY819704, *Sitobion avenae* Ace1); SaAce2 (AY707319, *S. avenae* Ace2); RpAce1 (AY667435, *Rhopalosiphum padi* Ace1); RpAce2 (AY707318, *R. padi* Ace2); DmAce (X05893, *Drosophila melanogaster* Ace); AgAce1 (XM_321792, *Anopheles gambiae* Ace1); AgAce2 (BN000067, *A. gambiae* Ace2); LdeAce1 (FJ647186, *Liposcelis decolor* Ace1); LdeAce2 (FJ647187, *L. decolor* Ace2); OvAce1 (FJ228227, *Orchesella villosa* Ace1); OvAce2 (FJ228228, *O. villosa* Ace2); LeAce1 (EU854149, *Liposcelis entomophila* Ace1); LeAce2 (EU854150, *L. entomophila* Ace2); BgAce1 (DQ288249, *Blattella germanica* Ace1); BgAce2 (DQ288847, *B. germanica* Ace2); BtAce1 (EF675188, *Bemisia tabaci* Ace1); BtAce2 (EF675190, *B. tabaci* Ace2); CqAce1 (XM_001847396, *Culex quinquefasciatus* Ace1); CqAce2 (XM_001842175, *C. quinquefasciatus* Ace2); BmoAce1 (NP_001037380 *Bombyx mori* Ace1); BmoAce2 (NP_001108113 *B. mori* Ace2); ApAce1 (XM_001948618, *Acyrthosiphon pisum* Ace1); ApAce2 (XM_001948953, *A. pisum* Ace2); NvAce1 (XM_001600408, *Nasonia vitripennis* Ace1); NvAce2 (XM_001605518, *N. vitripennis* Ace2); PhAce1 (AB266605, *Pediculus humanus corporis* Ace1); PhAce2 (AB266606, *P. humanus corporis* Ace2); CpAce1 (DQ267977, *Cydia pomonella* Ace1); CpAce2 (DQ267976, *C. pomonella* Ace2); HaAce1 (DQ001323, *Helicoverpa assulta* Ace1); HaAce2 (AY817736, *H. assulta* Ace2); AaAce1 (AB218421, *Aedes albopictus* Ace1); AaAce2 (AB218420, *A. albopictus* Ace2); AgoAce1 (AF502081, *Aphis gossypii* Ace1); AgoAce2 (AF502082, *A. gossypii* Ace2); CtAce1 (AB122151, *Culex tritaeniorhynchus* Ace1); CtAce2 (AB122152, *C. tritaeniorhynchus* Ace2); SgAce1 (AF321574, *Schizaphis graminum* Ace1); MpAce1 (AF287291, *Myzus persicae* Ace1); MpAce2 (AY147797, *M. persicae* Ace2); CpiAce1 (AJ489456, *Culex pipiens* Ace1); CpiAce2 (AM159193, *C. pipiens* Ace2); MdAce (AY134873, *Musca domestica* Ace); PxAce1 (AY970293, *Plutella xylostella* Ace1); PxAce2 (AY061975, *P. xylostella* Ace2); CsAce1 (EF453724, *Chilo suppressalis* Ace1); CsAce2 (EF470245, *C. suppressalis* Ace2); CmAce2 (FN538987, *Cnaphalocrocis medinalis* Ace2); PhcAce1 (AB266614, *Pediculus humanus capitis* Ace1); PhcAce2 (AB266615, *P. humanus capitis* Ace2); TuAce (AY188448, *T. urticae* Ace); AaeAce1 (EF209048, *Aedes aegypti* Ace1); LmAce1 (EU231603, *Locusta migratoria manilensis* Ace1); LbAce1 (FJ647185, *Liposcelis bostrychophila* Ace1); LbAce2 (EF362950, *L. bostrychophila* Ace2); NlAce (FM866396, *Nilaparvata lugens* Ace); CcAce (EU130781, *Ceratitis captitata* Ace); AmAce (AB181702, *Apis mellifera* Ace); BdAce2 (AY155500, *Bactrocera dorsalis* Ace2); HiAce (AY466160, *Haematobia irritans* Ace); CppAce1 (AY762905, *Culex pipiens pallens* Ace1); HarAce2 (AF369793, *Helicoverpa armigera* Ace2); BoAce (AF452052; *Bactrocera oleae* Ace); LcAce (U88631, *Lucilia cuprina* Ace); NcAce (AF145235, *Nephotettix cincticeps* Ace); LdAce2 (L41180, *Leptinotarsa decemlineata* Ace2).

The phylogenetic tree of the deduced amino acid sequences of AChEs from the Pacific electric ray (*Torpedo californica*), twospotted spider mite (*Tetranychus urticae*), and all the insect species available in GenBank was generated using the neighbor-joining method. Phylogenetic analysis suggests that there are two major groups (Ace1 and Ace2); TcAce1 and TcAce2 belong to the *Ace1*- and *Ace2*-type genes, respectively (Fig. 4). As expected, the cDNA-deduced TcAce1 has high protein sequence identities to BgAce1 (70%), HaAce1 (64%), AgAce1 (61%), SaAce1 (57%), and SgAce1 (57%). The cDNA-deduced TcAce2 has high protein sequence identities to LdAce2 (84%), HaAce2 (69%), BgAce2 (68%), AgAce2 (60%), SaAce2 (57%), and DmAce (55%) (Table 2). TcAce1 and TcAce2 exhibited 39% and 38% protein sequence identities to *T. californica* AChE, respectively. However, the protein sequence identities of Ace1 and Ace2 in the same insect species are 36% (between TcAce1 and TcAce2), 31% (between SaAce1 and SaAce2), 35% (between AgAce1 and AgAce2), 35% (between BgAce1 and BgAce2), and 32% (between HaAce1 and HaAce2) (Table 2).

**Table 2:** Percent identities of amino acid residues among the AChEs of *Tribolium castaneum*, *Torpedo californica* and other seven insect species.

Name	Tca	TcAce1	SaAce1	AgAce1	BgAce1	SgAce1	HaAce1	DmAce	TcAce2	SaAce2	AgAce2	BgAce2	LdAce2	HaAce2
Tca	—	39	38	42	40	38	39	35	38	37	37	38	37	37
TcAce1		—	57	61	70	57	64	31	36	31	34	36	34	34
SaAce1			—	51	59	96	55	32	36	31	33	34	33	34
AgAce1				—	57	51	59	34	37	33	35	39	35	36
BgAce1					—	59	62	33	36	33	33	35	36	34
SgAce1						—	55	32	36	30	33	34	34	33
HaAce1							—	31	35	30	32	35	34	32
DmAce								—	55	48	62	51	53	51
TcAce2									—	57	60	68	84	69
SaAce2										—	50	53	55	52
AgAce2											—	55	57	56
BgAce2												—	63	60
LdAce2													—	66
HaAce2														—

NOTE: TcaAce, *Torpedo californica*; TcAce, *Tribolium castaneu*m; SaAce, *Sitobion avenae*; DmAce, *Drosophila melanogaster*; AgAce, *Anopheles gambiae*; BgAce, *Blattella germanica*; HaAce, *Helicoverpa assulta*; SgAce, *Schizaphis graminum*; LdAce, *Leptinotarsa decemlineata*.

It is worth noting that, per the present nomenclature, the reported PhAce1, PhcAce1, AgoAce1, MpAce1 and CtAce1 in the Ace2 group should change to PhAce2, PhcAce2, AgoAce2, MpAce2 and CtAce2, respectively, whereas the reported PhAce2, PhcAce2, AgoAce2, MpAce2 and CtAce2 in the Ace1 group should be named PhAce1, PhcAce1, AgoAce1, MpAce1 and CtAce1, respectively (Fig. 4).

### Three-dimensional models

#### TcAce1 (TcAP-AChE)

**Figure 5:**
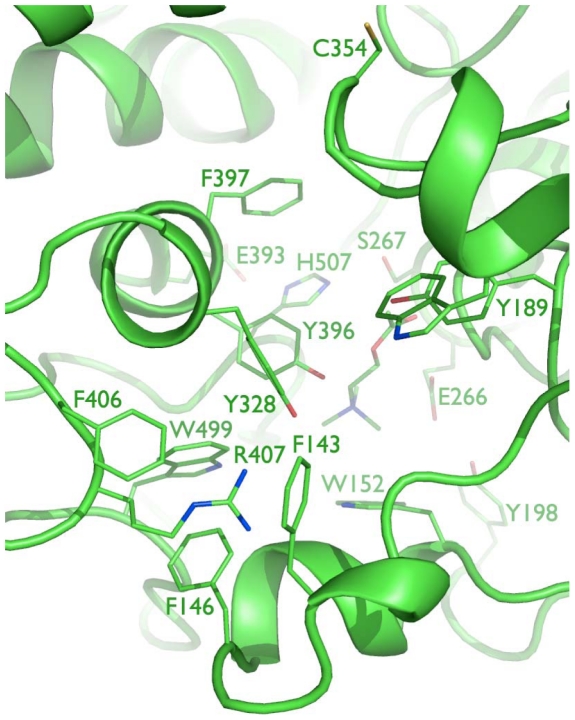
Close-up view of the active site of TcAce1 with a perspective from the free cysteine at the opening of the active-site gorge down to ACh and the catalytic triad at the bottom of the gorge.

**Figure 6:**
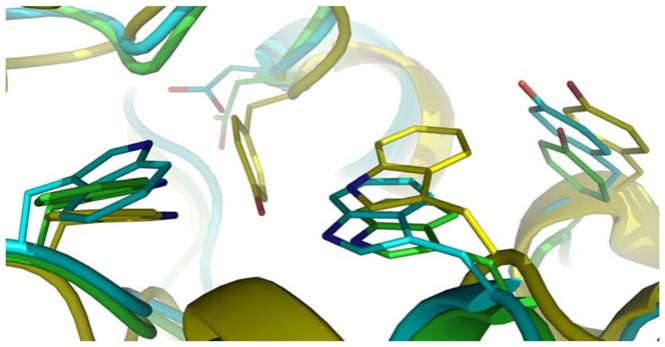
Comparison of the bottom of the active-site gorge in TcAce1 to those in human AChE and *Anopheles gambiae* AP-AChE. Tyr449 in human AChE (yellow) is mutated to Asp509 in TcAce1 (green) and Asp441 in *A. gambiae* AChE (cyan).

Eighteen 10-ns molecular dynamics simulations of the substrate-bound TcAce1 protein homology model derived from a human butyrylcholinesterase (hBChE) crystal structure (Protein Data Bank ID: 2J4C [42]) resulted in a time-averaged model with a distorted catalytic triad and partial unfold of the omega loop. This result indicates that the homology model is structurally unstable, presumably due to the low sequence identity of the omega loop between TcAce1 and hBChE that results in gaps in the omega loop. Another homology model was therefore built from a simulation-refined model of the African malaria mosquito (*A. gambiae*) AP-AChE (Protein Data Bank ID: 2AZG) [33] that has a higher sequence identity to TcAce1 (73%) than the identity between hBChE and TcAce1 (46%). Twenty-two 10-ns molecular dynamics simulations of this model liganded with its substrate yielded a time-averaged model without distortions in the catalytic triad and the omega loop. In this final model of TcAce1 (Fig. 5), C354 is exposed to solvent and accessible to covalent bonding at the opening of the active-site gorge [33]; R407 is enclosed by F143, F146, and F406 via cation-pi interactions; Y189, Y396, and Y400 adopt conformations that make the gorge relatively open; ACh adopts the fully extended conformation with its ammonium group forming a cation-pi interaction with the indole ring of W152 and its carbonyl oxygen atom anchored at the oxyanion hole comprising of G186, G187, and A268; E266 forms a hydrogen bond with Y198 at the bottom of the active-site gorge; E393, H507, and S267 form the catalytic triad. The TcAce1 active site is very similar to those of *A. gambiae* and *S. graminum* AP-AChEs [33], [34], and different from the human AChE active site in that Y449 in the human enzyme is replaced by Asp creating void space at the bottom of the TcAce1 active site (Fig. 6). In *T. californica* AChE, rotation of Y442, which corresponds to Y449 of human AChE, reportedly controls the opening of a 3.4-Å-wide channel that enables rapid clearance of substrate hydrolysis products [43]. In DmAChE, the counterpart of Y449 is also mutated to Asp; hence the crystal structure of DmAChE (Protein Data Bank ID: 1DX4 [41]) has a channel with a diameter of ∼5 Å that is formed by G79, W83, W472, L479, and D482 and connects the active-site gorge to solvent [44]. Because of the mutation Tyr of human to Asp of TcAce1, the final model of TcAce1 has a similar channel that is comprised of G148, W152, W499, M506, and D509. The TcAce1 channel is, however, partially blocked by M151 and P498.

#### TcAce2 (TcAO-AChE)

A homology study identified the crystal structure of DmAChE (PDB ID: 1DX4 [41]) as a template with a sequence identity of 60% and generated a homology model of TcAce2. This model has a protruded large loop conformation for residues 145–162, which is due to the omission of the corresponding loop (residues 103–136) in the 1DX4 crystal structure. An initial set of 21 10-ns molecular dynamics simulations of the TcAce2 homology model without residues 145–162 resulted in a time-average model with the catalytic triad distorted.

In the loop of residues 145–162, there are two histidine residues, four arginine residues, two lysine residues, one aspartate residue, and one glutamate residue. At the physiological pH of 7.4, this loop has a net charge of +4. To avoid a possible effect of the highly-charged loop on the catalytic triad distortion, a second set of 21 10-ns simulations of the TcAce2 homology model possessing residues 145–162 was performed. The triad was still distorted in an average conformation of all trajectories saved at 1.0-ps intervals during the last 1-ns period of all 21 simulations or in an average of each cluster of the trajectories generated by a cluster analysis. As to the loop conformation of residues 145–162, the cluster analysis showed that 24% of the trajectories had residues 145–162 folded in contact with surface residues such as R21, D148, D501, and D203.

A third set of 22 10-ns simulations of TcAce2 with residues 145–162 adopting the folded conformation was then carried out. The initial conformation of the third set of simulations was obtained from averaging all the trajectories of TcAce2 with the folded loop conformation of residues 145–162 followed by manual adjustment of the side-chain torsions to restore the hydrogen bond network of the catalytic triad. A cluster analysis of all the trajectories saved at 1.0-ps intervals during the last 1.0-ns period of all 22 simulations showed that residues 145–162 remain the folded conformation. However, the average of all the trajectories had a distorted catalytic triad. Visual inspection of all 22 simulations found that conformations of the first of the 22 simulations have a catalytic triad engaging in a hydrogen-bond network. The final model of TcAce2 was then obtained from averaging all trajectories saved at 1.0-ps intervals during the last 1.0 ns period of the first simulation.

**Figure 7:**
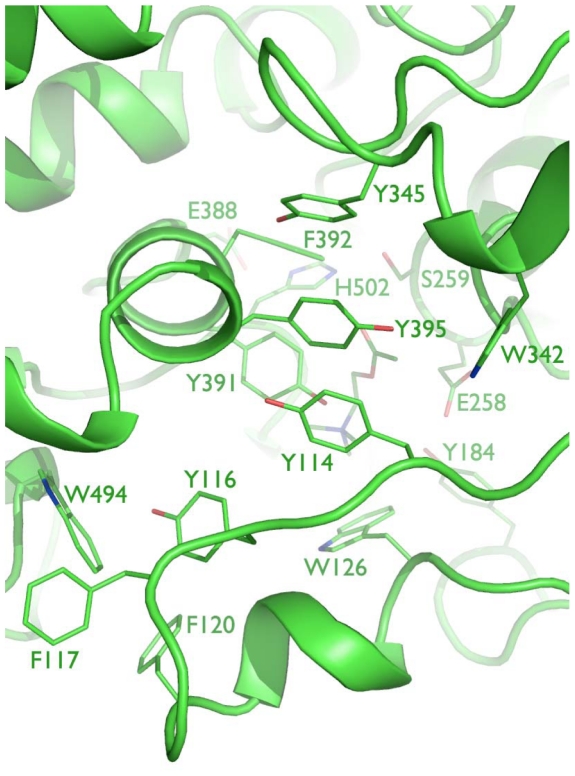
Close-up view of the active site of TcAce2 with a perspective looking down to acetylcholine and the catalytic triad at the bottom of the gorge.

**Figure 8:**
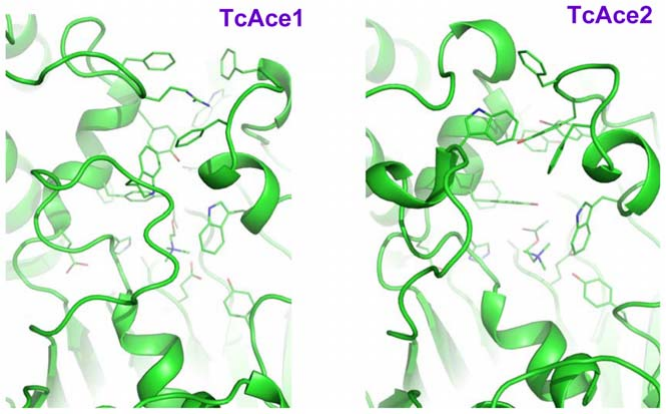
Comparison of loop conformations of residues 146–154 and 493–509 in TcAce1 with the corresponding ones of residues 120–128 and 488–504 in TcAce2.

Although the homology model of TcAce2 was based on the DmAChE crystal structure, the active site of the simulation-refined TcAce2 model (Fig. 7) is very different from that of the DmAChE crystal structure. It is also different from those of human AChE and insect AP-AChEs (*i.e.*, AChE1s). In the refined model of TcAce2 (Fig. 7), Y114, Y345, Y395, and W342 form an aromatic cluster that completely block the entrance of the active site; E388, H502, and S259 form a catalytic triad; ACh has a cation-pi interaction with W126, but it does not adopt the fully extended conformation, nor is its carbonyl oxygen atom located in the oxyanion hole. In contrast to the TcAce1 model with residues 146–154 and 493–509 that partially shield ACh from interacting with solvent, the TcAce2 model has its corresponding residues (120–128 and 488–504) adopt conformations that leave ACh to be exposed to solvent (Fig. 8).

### *TcAce* gene expression profiles

**Figure 9:**
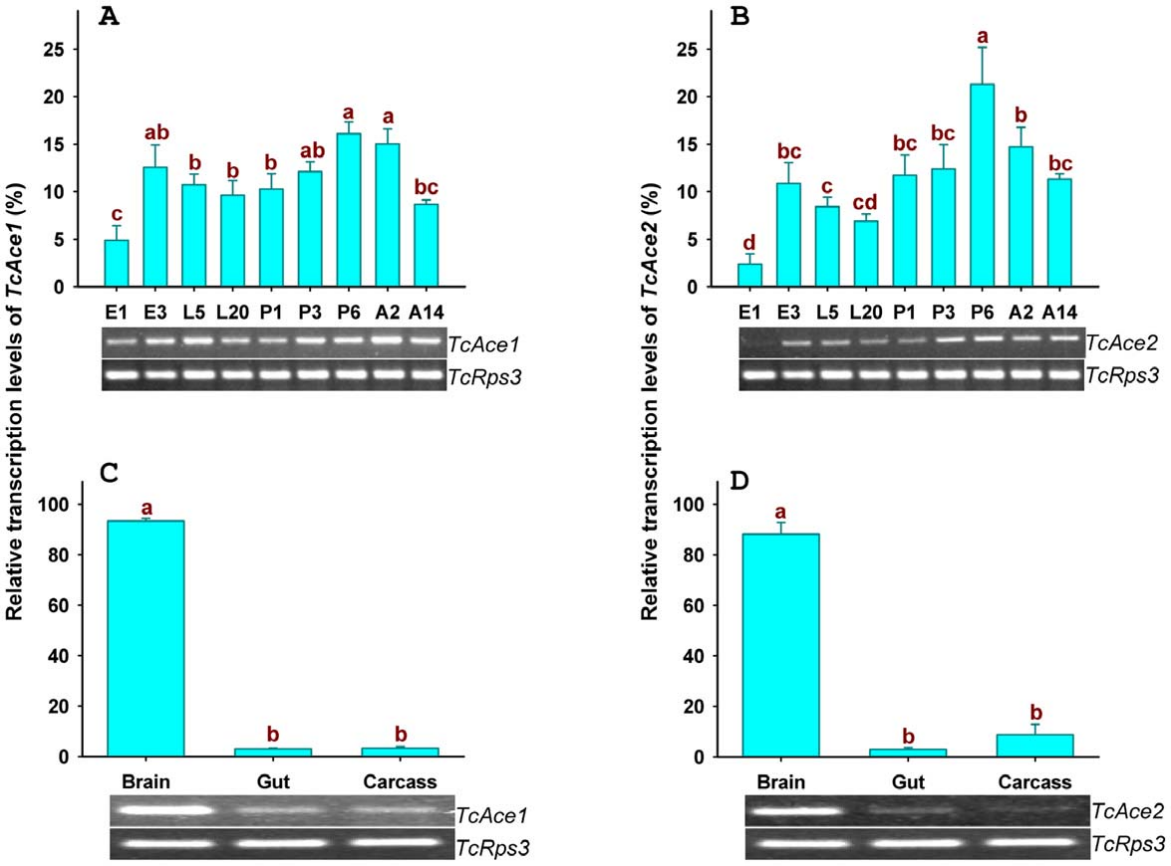
The two *TcAce* genes mRNA expression levels of developmental stages and different tissues were determined by RT-PCR as shown by gel pictures at the bottom of each panel and quantitative real-time PCR as shown by histograms. Real-time PCR data were normalized to *TcRps3* gene expression. E1, 1-d eggs; E3, 3-d eggs; L5, 5-d larvae; L20, 20-day larvae; P1, 1-d pupae; P3, 3-d pupae; P6, 6-d pupae; A2, 2-d adults; A14, 14-d adults. Gut (including midgut and hindgut), Carcass (not including head, gut and nerve system). Standard error bars were base on three replicates. One-Way ANOVA-Fisher's LSD was used in statistical analysis of quantitative real-time PCR data.

The transcript levels of *TcAce1* and *TcAce2* were evaluated by reverse transcription PCR (RT-PCR) and quantitative real-time PCR (qPCR) in tissues of *T. castaneum* at different developmental stages (Fig. 9). Both *TcAce* genes were transcribed in all the stages examined, including 1-day (d) and 3-d eggs; 5-d and 20-d larvae; 1-d, 3-d and 6-d pupae; and *2*-d and 14-d adults. The lowest expression levels of these genes were found in eggs, particularly for *TcAce2* whose expression level was undetectable by RT-PCR in 1-d eggs (Fig. 9A and 9B). The expression patterns of *TcAce1* and *TcAce2* were very similar. In addition, the *TcAce1* and *TcAce2* genes also exhibited similar tissue-specific expression patterns (Fig. 9C and 9D). As expected, these genes were predominately expressed in the brain, although their expressions were also detected in the gut and carcass after the brain and ventral nerve cord were removed.

## Discussion

Since the first insect AChE orthologous gene (*i.e.*, the one later named *Ace2* or AO-Ace) and the first insect AChE paralogous gene (*i.e.*, the one later named *Ace1* or AP-Ace) were reported in *D. melanogaster* in 1986 [9] and in *S. graminum* in 2002 [10], respectively, cDNAs encoding both *Ace1* and *Ace2* have been sequenced from each of at least 27 insect species. However, the gene models and the genomic organizations of *Ace1* and *Ace2* have not been well established in insects. In this study, we confirmed the two *Ace* gene (*TcAce1* and *TcAce2*) models in *T. castaneum* by sequencing the coding regions of their cDNAs followed by comparative analyses of their cDNA and genomic sequences. The *TcAce1* and *TcAce2* genes are significantly different not only in the length of their genomic DNA (*TcAce1* with 2,986 bp and *TcAce2* with 32,243 bp) but also in the intron/exon organizations. Specifically, *TcAce1* possesses only one intron whereas *TcAce2* has five introns. Furthermore, *TcAce1* is located on chromosome 5 (ChLG5), whereas *TcAce2* is on chromosome 2 (ChLG2; Fig. 3). Apparently, the intron/exon organizations and the chromosomal locations of these genes in *T. castaneum* are different from their counterparts in other insect species [8], [14], [18], [23], [45].

Despite the significant differences in genomic structures and the chromosomal locations of *TcAce1* and *TcAce2*, the deduced protein sequences of the two genes exhibit all the common features of an AChE sequence wise. These features include (1) a conserved active-site triad, including S267 in TcAce1 and S259 in TcAce2 (S200 in *Torpedo*), E393 in TcAce1 and E388 in TcAce2 (E327 in *Torpedo*), and H507 in TcAce1 and H502 in TcAce2 (H440 in *Torpedo*); (2) a choline binding site, W152 in TcAce1 and W126 in TcAce2 (W84 in *Torpedo*); (3) three pairs of cysteines putatively forming intramolecular disulfide bonding (C135∼C162, C321∼C334, and C469∼C591 in TcAce1; and C109∼C136, C313∼C328, and C464∼C580 in TcAce2); (4) a cysteine forming intermolecular disulfide bonding (C617 in TcAce1 and C600 in TcAce2); (5) 10 conserved aromatic amino acid residues out of 14 aromatic residues lining the catalytic gorge of AChE; and (6) the conserved sequence FGESAG, flanking S267 in TcAce1 and S259 in TcAce2 (Fig. 2).

Ethanolmine and glucosamine residues are characteristic of a C-terminal glycolipid anchor in most G2 AChE [46], [47]. Our analysis by using PredGPI predictor suggests that DmAChE and TcAce1 contain a GPI-anchor at the C-terminal. Although TcAce2 has higher sequence identity level with DmAChE than TcAce1, TcAce2 does not appear to contain a GPI-anchor at the C-terminal. Because the C-terminal Cys residue in AChE is reportedly for the intermolecular disulfide linkage [48], C617 in TcAce1 and C600 in TcAce2 are likely involved in the intermolecular disulfide linkage, although the corresponding Cys residue is missing in DmAChE [46]. We also analyzed the hydrophilic and hydrophobic of non-homologous amino acid residues of TcAce1 and TcAce2 and compared homologous sequences of TcAce1 and TcAce2 with mAChE and DmAChE at the C-terminal for possible formation of the four-helix bundle. Our analysis suggests that C-terminal sequences of both TcAce1 and TcAce2 may form the dimeric four-helix bundle.

According to comparisons of TcAce1 and TcAce2 with Ace proteins from other insect species, TcAce1 showed high sequence identities to the AChE1 (AP-AChE) proteins from *Sitobion avenae* (57%), *A. gambiae* (61%), *Blattella germanica* (70%), *S. graminum* (57%), and *Helicoverpa assulta* (64%; Table 2). Similarly, TcAce2 also showed high amino acid identities to the AChE2 (AO-AChE) proteins of *S. avenae* (57%), *D. melanogaster* (55%), *A. gambiae* (60%), *B. germanica* (68%), *Leptinotarsa decemlineata* (84%), and *H. assulta* (69%). Based on our analysis of deduced amino acid sequences from the two genes among respective insect species, the sequence identity levels within the paralogous (*Ace1*) or orthologous (*Ace2*) genes range from 48 to 96%, whereas the sequence identity levels between the *Ace1* and *Ace2* genes of the same insect species range only from 31 to 36% in all insect species examined (Table 2). These results support the hypothesis that the two *Ace* genes were originated from an old duplication before the diversification of insect species [32].

Furthermore, a phylogenetic tree, which was generated from the highly conserved regions of all insect and *T. urticae* AChE amino acid sequences available in GenBank and the corresponding one in *T. californica* using the neighbor-joining method, revealed two insect AChE clusters. *TcAce1* was grouped into the insect *Ace1* cluster and *TcAce2* into the insect *Ace2* cluster. The significant divergence between *TcAce1* and *TcAce2* suggests that these genes were evolved from their corresponding *Ace* gene lineages in insect species [49]. These results also suggest, for the first time, that the divergence of *Ace1* and *Ace2* might occur prior to insect speciation and that the *Ace1* gene might be lost in *D. melanogaster* and other species in Cyclorrapha suborder of Diptera during the evolutionary process [14], [25], [32]. Thus, it is likely that both the *Ace* genes in insects may have different functions because these genes have evolved during the evolutionary histories of these insect species.

Using a reported simulation-refined model of *A. gambiae* AP-AChE [33] and a crystal structure of DmAChE [41] with high sequence identities to TcAce1 (73%) and TcAce2 (60%), respectively, and the same multiple molecular dynamics simulation method to model and refine TcAce1 and TcAce2, we obtained models of TcAce1 and TcAce2 both which are in complex with acetylcholine. The TcAce1 model has an active site that is almost identical to those of *A. gambiae* and *S. graminum* AP-AChEs, and it has C354 at the opening of the active-site gorge just like the insect-specific C286 of *A. gambiae* and C289 of *S. graminum* that are susceptible to sulfhydryl agents [33], [34]. The TcAce2 model has an active site with an entrance comprised of G122, W126, W494, M501, and D504. This entrance corresponds to the small opening at the bottom of the active-site gorge of DmAChE [44] or *T. californica* AChE when Y442 moves away from W84 [43]. In the TcAce2 model, the region that corresponds to the entrance of TcAce1 is completely blocked by Y114, Y345, Y395, and W342. Of the four aromatic residues, Y395 and W342 correspond to Y334 and W279 of *T. californica* AChE, respectively, and belong to the 14 conserved aromatic residues that line the active-site gorge of *T. californica* AChE [50]. In other words, the entrance of the active-site gorge of TcAce2 appears to be reversed relative to that of TcAce1. Unlike ACh in the TcAce1 model, ACh does not adopt the fully extended conformation and its carbonyl oxygen atom is not placed in the oxyanion hole in the TcAce2 model.

**Figure 10:**
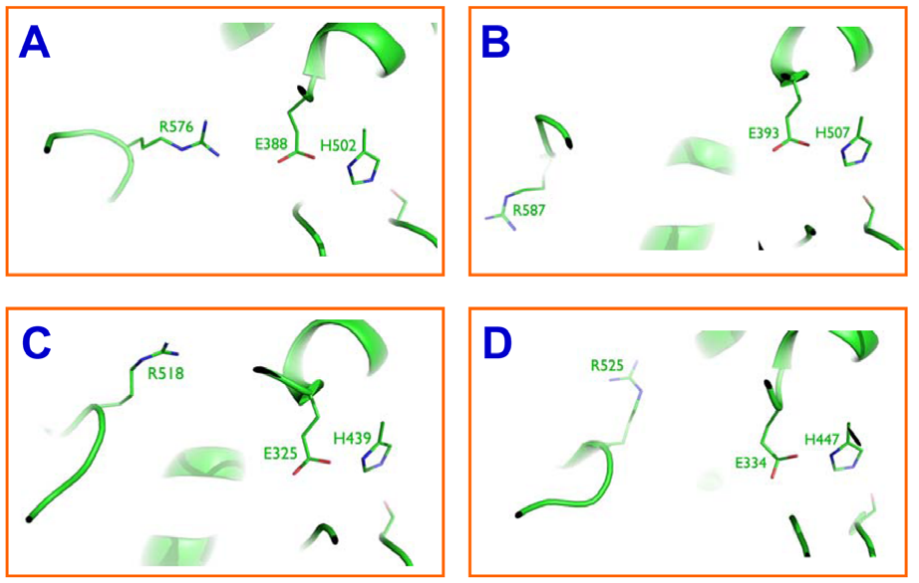
Separation of Arg from the catalytic triad in TcAce2 (A), TcAce1 (B), *Anopheles gambiae* AP-AChE (C), and human AChE (D).

In addition, R576 in the TcAce2 model is close to E388 (the separation between the side-chain N atom of R576 and the side-chain O atom of E388 is 3.9 Å), a composite residue of the catalytic triad, in contrast to the corresponding arginine residue that is away from the catalytic glutamate residue in TcAce1, *A. gambiae* AP-AChE and human AChE (Fig. 10). Analysis of all the trajectories of the third set of simulations of TcAce2 showed that 95% of the trajectories has a hydrogen bond between R576 and E388, accompanied by hydrogen bonds between H502 and E505 and between S259 and E258, leading to disruption of the catalytic triad. These computational observations suggest that TcAce1 is a robust ACh hydrolase and susceptible to sulfhydryl agents and that TcAce2 is not a catalytically efficient ACh hydrolase, although further study is needed to comprehensively elucidate physiological functions of *Ace1* and *Ace2* genes. In view of these computational results, it is logical to investigate whether TcAce2 functions more as a cholinesterase-like adhesion molecule (CLAM) [51], [52] than TcAce1. However, our sequence analysis using ClustalW 2.0.12 shows that TcAce1 has a slightly higher sequence homology to *D. melanogaster* gliotactin, which is one of the three *D. melanogaster* CLAMs, than TcAce2 (Table 3); both TcAce1 and TcAce2 have dipole moments that are comparable to those of other AChEs (Table 4). The orientations of the dipole moments of TcAce1 and TcAce2 are almost the same. The two dipole moments are approximately along the beta strand that corresponds to Strand 5 of the *T. californica* AChE crystal structure [53] and nearly identical to those of other AChEs but orthogonal to that of *Galactomyces geotrichum* lipase [52], [54]. These sequence and dipole moment analyses do not support the hypothesis that TcAce2 functions as a CLAM.

**Table 3:** Percentages of amino acid sequence identity of TcAce1 and TcAce2 to cholinesterase-like lipase and adhesion proteins.

AChE	*Gc*NeutralLipase	*Dm*Neuroligin	*Dm*Neurotactin	*Dm*Gliotactin
*Torpedo californica* AChE	23	19	17	25
TcAChE1	19	21	17	27
TcAChE2	20	20	18	25

The NCBI session numbers of *Gc*NeutralLipase, *Dm*Neuroligin, *Dm*Neurotactin, and *Dm*Gliotactin are P79066, AAF52450, CAA37831, and AAC41579, respectively.

**Table 4:** The electrostatic characteristics of TcAce1, TcAce2, and other proteins.

PDB ID (Species)	Total atoms	Total residues	Net charge of N terminal deletion (*e*)	Net charge of C terminal deletion (*e*)	Net charge of deletion between N and C termini (*e*)	Net charge of the structure or model (*e*)	Dipole moment (Debye)	Dipole moment per atom (Debye)
TcAce1 (*Tribolium castaneum, AP*)	4201	529	−5	0	0	−3	1490	0.35
TcAce2 (*Tribolium castaneum, AO*)	4353	545	−1	+1	0	−19	1163	0.27
1QO9 (*Drosophila melanogaster, AO*)	4273	540	0	−2	+1	−18	669	0.16
2AZG (*Anopheles gambiae, AP*)	4243	536	0	−1	0	−8	1718	0.40
2HCP (*Schizaphis graminum, AP*)	4302	540	−7	+2	0	−8	1312	0.30
2ACE (*Torpedo californica*)	4143	527	−2	−1	−1	−8	1819	0.44
1J06 (*Mus musculus*)	4177	535	+2	−2	0	−9	867	0.21
2X8B (*Homo sapiens*)	4179	536	0	−2	0	−10	1384	0.33
1THG (*Galactomyces geotrichum*, lipase)	4287	543	+1	0	0	−15	814	0.19

## Materials and Methods

### Insect culture

The Georgia-1 (GA-1) strain of *T. castaneum* was reared on whole-wheat flour containing 5% (by weight) of brewers' yeast at 30°C and 65% relative humidity under standard conditions in the laboratory of Kansas State University (Manhattan, Kansas, United States of America) based on the method of Haliscak and Beeman [55].

### Total RNA isolation and reverse transcription

Total RNA was isolated from *T. castaneum* samples using TRIzol reagent following the recommended procedure by Invitrogen (Carlsbad, California, United States of America). The RNA was treated with DNase I (Fermentas, Glen Burnie, Maryland, United States of America) according to the manufacturer's instruction and the first-strand cDNA template was synthesized from 3.0 µg of total RNA by using First Strand cDNA Synthesis Kit (Fermentas) with oligo (dT)_18_ as the primer.

### Subcloning and sequencing of cDNA

To obtain the cDNAs corresponding to the entire protein coding regions of *TcAce1* and *TcAce2*, we designed specific primers based on *TcAce* gene predictions and their genomic organization (Table 1). The PCR products of each reaction were subjected to electrophoresis on 1% agarose gel containing ethidium bromide. The PCR bands were excised and purified using QIAEX II Agarose Gel Extraction Kit (Qiagen, Valencia, California, United States of America). The purified fragment was subcloned into a pGEM-T Easy Vector (Invitrogen) according to the manufacturer's instruction. The ligation DNA mixtures were used to transform bacterial cells by using Z-Competent *E. coli* Transformation Kit and Buffer Set™ (Zymo Research Corporation, Irvine, California, United States of America). Plasmids were isolated from the bacterial cells and used for DNA sequencing (KSU DNA Sequencing and Genotyping Facility, Manhattan, Kansas, United States of America). Signal P software was used to predict signal peptide [56].

### Phylogenetic analysis of AChEs

ClustalW software (www.ebi.ac.uk/clustalw/) [57], was used to perform multiple sequence alignments prior to phylogenetic analysis. Phylogenetic analysis was done using MEGA 4.0 [58] for construction a neighbor-joining tree to examine the evolutionary relationships among *T. californica*, *T. urticae* and 43 insect species. To evaluate the branch strength of the phylogenetic tree, a bootstrap analysis of 1000 replications was performed.

### AChE model prediction

#### Model preparation

The starting conformations of TcAce1 and TcAce2 used in the multiple molecular dynamics simulations were generated by the SWISSMODEL homology program using a computer model (Protein Data Bank ID: 2AZG [33]) and a crystal structure (Protein Data Bank ID: 1DX4 [41]) as templates, respectively. ACh was manually docked into the active site of TcAce1 or TcAce2 according to the bound ACh conformation in the crystal structure of *T. californica* AChE (Protein Data Bank ID: 2ACE [59]). All His, Glu, Asp, Arg, and Lys residues of the ACh-bound TcAce1 or TcAce2 were treated as HIP, GLU, ASP, ARG, and LYS, respectively. The topology and coordinate files were generated by the PREP, LINK, EDIT, and PARM modules of the AMBER 5.0 program [60]. The complex was refined by energy minimization using the SANDER module of the AMBER 5.0 program with a dielectric constant of 1.0 and 500 cycles of steepest-descent minimization followed by 10,000 cycles of conjugate-gradient minimization. The energy-minimized ACh complex with TcAce1 or TcAce2 was solvated with 5,897 or 6,744 TIP3P water molecules [61], leading to a system of 20,703 or 23,244 atoms, respectively. The water molecules were obtained from solvating the complex using a pre-equilibrated box of 216,000 TIP3P molecules, whose hydrogen atom charge was set to 0.4170, where any water molecule was removed if it had an oxygen atom closer than 2.2 Å to any solute atom or a hydrogen atom closer than 2.0 Å to any solute atom, or if it was located further than 10.0 Å along the x-, y-, or z-axis from any solute atom.

#### Multiple molecular dynamics simulations

The solvated protein complex was energy-minimized for 100 cycles of steepest-descent minimization followed by 100 cycles of conjugate-gradient minimization to remove close van der Waals contacts in the system, then heated from 0 to 300 K at a rate of 10 K/ps under constant temperature and volume, and finally simulated independently with a unique seed number for initial velocities at 300 K under constant temperature and pressure using the PMEMD module of the AMBER 8.0 program [62] with the AMBER force field (ff99SB) [63], [64]. All simulations used (1) a dielectric constant of 1.0, (2) the Berendsen coupling algorithm [65], (3) a periodic boundary condition at a constant temperature of 300 K and a constant pressure of 1 atm with isotropic molecule-based scaling, (4) the Particle Mesh Ewald method to calculate long-range electrostatic interactions [66], (5) a time step of 1.0 fs, (6) the SHAKE-bond-length constraints applied to all the bonds involving the H atom, (7) saving the image closest to the middle of the “primary box” to the restart and trajectory files, (8) formatted restart file, and (9) default values of all other inputs of the PMEMD module. All simulations were performed on a cluster of Apple Mac Pros with 80 Intel Xeon cores (3.0 GHz) and a cluster of Apple Xserves with 590 G5 processors (2.2/2.3 GHz).

#### Simulation analysis

Average structures were obtained by using the CARNAL module of AMBER 5.0. Cluster analyses were performed by using the PTRAJ module [67] of AMBER 10.

#### Dipole moment calculations

All dipole moment calculations were performed using the Protein Dipole Moments Server (http://bioinfo.weizmann.ac.il/dipol/indexj.html) [68]. Protein structures with minimal deletions were used and ligands and structural water molecules were removed before the dipole moment calculations.

### Analysis of expression of *TcAce1* and *TcAce2* by RT-PCR and qPCR

The expression patterns of both *TcAce1* and *TcAce2* genes were analyzed at various developmental stages including embryos (1 day and 3 days eggs), early larvae (5 days larvae), late larvae (20 days larvae), early pupae (1 day pupae), middle pupae (3 days larvae), late pupae (6 days pupae), early adults (2 days adults) and two weeks old adults. To analyze tissue specific expression, we collected samples from the following dissected late pupa tissues (pooled from thirty late pupae): brain, gut (midgut and hind gut) and carcass (the whole body excluding brain, ganglia and gut). For all the samples, 3.0 µg of total RNA were treated with DNase I (Fermentas) to remove any genomic DNA contaminations, and then used as templates for the first strand cDNA synthesis. The cDNAs prepared from total RNA were used as templates for amplification and detection of specific *TcAce* sequences. The gene-specific primers were designed by using the Beacon Designer 2.0 software (Premier Biosoft International, Palo Alto, California, United States of America) and are shown in Table 1. For reverse transcription PCR (RT-PCR), cDNA fragments of each *TcAce* were amplified using the PCR conditions as follows: 94°C for 1.5 min followed by 30 cycles (26 cycles for *TcRps3* gene) of 94°C 30 s, 55°C 30 s and 72°C 45 s. A final extension at 72°C for 5 min was added at the end of the PCR. The relative mRNA expression of each *TcAce* was assessed by qRT-PCR using SYBR-Green in the Bio-Rad iCycler iQ™ multi-coclor real-time PCR detection system (Bio-Rad Laboratories, Hercules, CA, USA) based on the method of Giulietti et al. [69]. All the experiments were performed in triplicate and normalized to the mRNA level of ribosomal protein S3 (*Rps3*) as a reference gene for each sample [70]. The relative mRNA expression levels were calculated according to the 2^−ΔΔCt^ method [71].

### Statistical analysis

The data from the qPCR analysis were subjected to ANOVA followed by Fisher's least significant difference (LSD) multiple comparisons to separate the means among the treatments by using ProStat software (Poly Software International, Pearl River, New York, United States of America).
